# The Cerebellar Cognitive Affective/Schmahmann Syndrome: a Task Force Paper

**DOI:** 10.1007/s12311-019-01068-8

**Published:** 2019-09-14

**Authors:** Georgios P. D. Argyropoulos, Kim van Dun, Michael Adamaszek, Maria Leggio, Mario Manto, Marcella Masciullo, Marco Molinari, Catherine J. Stoodley, Frank Van Overwalle, Richard B. Ivry, Jeremy D. Schmahmann

**Affiliations:** 1 Nuffield Department of Clinical Neurosciences, University of Oxford, Oxford, UK; 2 Rehabilitation Research Center REVAL, UHasselt, Hasselt, Belgium; 3 Clinical and Cognitive Neurorehabilitation, Center of Neurology and Neurorehabilitation, Klinik Bavaria Kreischa, An der Wolfsschlucht 1-2, 01703 Kreischa, Germany; 4 Department of Psychology, Sapienza University of Rome, Rome, Italy; 5 Ataxia Laboratory, IRCCS Fondazione Santa Lucia, Rome, Italy; 6 Department of Neurology, CHU-Charleroi, 6000 Charleroi, Belgium; 7 Department of Neurosciences, University of Mons, 7000 Mons, Belgium; 8 SPInal REhabilitation Lab (SPIRE), IRCCS Fondazione Santa Lucia, Via Ardeatina 306, 00179 Rome, Italy; 9 Neuro-Robot Rehabilitation Lab, IRCCS Fondazione Santa Lucia, Via Ardeatina 306, 00179 Rome, Italy; 10 Department of Psychology, American University, Washington, DC 20016 USA; 11 Department of Psychology, Vrije Universiteit Brussel, Brussels, Belgium; 12 Department of Psychology, University of California, Berkeley, CA USA; 13 Ataxia Unit, Cognitive Behavioral Neurology Unit, Laboratory for Neuroanatomy and Cerebellar Neurobiology, Department of Neurology Massachusetts General Hospital, Harvard Medical School, Boston, MA USA

**Keywords:** Cerebellum, Cognition, Emotion, Affect, Cerebellar cognitive affective syndrome, Schmahmann syndrome

## Abstract

Sporadically advocated over the last two centuries, a cerebellar role in cognition and affect has been rigorously established in the past few decades. In the clinical domain, such progress is epitomized by the “cerebellar cognitive affective syndrome” (“CCAS”) or “Schmahmann syndrome.” Introduced in the late 1990s, CCAS reflects a constellation of cerebellar-induced sequelae, comprising deficits in executive function, visuospatial cognition, emotion–affect, and language, over and above speech. The CCAS thus offers excellent grounds to investigate the functional topography of the cerebellum, and, ultimately, illustrate the precise mechanisms by which the cerebellum modulates cognition and affect. The primary objective of this task force paper is thus to stimulate further research in this area. After providing an up-to-date overview of the fundamental findings on cerebellar neurocognition, the paper substantiates the concept of CCAS with recent evidence from different scientific angles, promotes awareness of the CCAS as a clinical entity, and examines our current insight into the therapeutic options available. The paper finally identifies topics of divergence and outstanding questions for further research.

## Introduction: the Cerebellar Cognitive Affective/Schmahmann Syndrome (G.P.D. Argyropoulos, K. van Dun, and M. Manto)

A series of sporadic investigations in the last two centuries had advocated the need to revise the confinement of cerebellar (CB) function to the motor domain [1]. The rediscovery of this proposal and its systematic investigation over the last few decades (see [2] for a review) have now firmly established a CB role in cognition and affect.

One of the fundamental frameworks attempting to explain these contributions was the “dysmetria of thought hypothesis” [3–5, 11], whereby a “universal cerebellar transform” (UCT) is applied over multiple functional domains. The proposal was predicated on: (i) the CB cytoarchitectural homogeneity, implying the implementation of a unitary computation by the cortico-nuclear microcomplex, the fundamental CB computational unit [6, 7]; (ii) the functional CB heterogeneity, given the modular specificity of multiple cerebro-CB anatomical connections through the feedforward (cortico-ponto-CB) [8], and the feedback (CB-thalamo-cortical) limbs [9] that bring it in close interaction with supratentorial motor, paralimbic, and association cortices; (iii) the well-defined motor syndromes following damage to lobules communicating with motor and premotor cortices [10]. Disruptions of CB components of the nonmotor cerebro-CB circuits were hypothesized to deprive cognitive and affective processes from the UCT, producing an impairment in the coordination of thought, similar to the impairments in motor coordination that are the hallmark of ataxia.

Shortly following the formulation of the “dysmetria of thought” hypothesis [11], Schmahmann and Sherman reported a complex pattern of cognitive and affective deficits characterizing a group of 20 patients with focal CB lesions. This entity was termed “cerebellar cognitive affective syndrome” (CCAS) [12], also referred to as “Schmahmann syndrome” [13]. On the basis of neurological examination, bedside mental state tests, and neuropsychological assessment, CCAS was proposed to reflect a constellation of CB-induced sequelae, comprising deficits in (i) executive function: impaired working memory (e.g., deficient mental arithmetic), set-shifting, verbal fluency (manifesting as telegraphic speech, unrelated to dysarthria), problem-solving, multitasking, planning, sequencing, and organizing activities; (ii) visuospatial cognition: visuospatial disintegration (manifesting as a deficit in copying and conceptualizing drawn figures) and simultanagnosia; (iii) language, over and above speech: agrammatism, mild anomia, and dysprosodia; and (iv) emotion–affect: flattening of affect or disinhibition (often manifesting as humorous yet inappropriate comments, impulsive actions, and overfamiliarity), regressive and childlike behavior in some patients and obsessive–compulsive traits in others, and pathological laughing and crying [12]. Further insight has more recently been gained from a neuropsychiatric perspective by the identification of five core features: deficits in attentional or emotional control, autism or psychosis spectrum symptoms, and deficient social skills. The symptoms within each domain were conceptualized as reflecting either exaggerated-hypermetric or diminished-hypometric responses to the internal and/or external environment [14].

Overall, these symptoms were attributed to disruptions of pathways reciprocally connecting the CB with limbic circuitry and prefrontal, temporal, and parietal association cortices. More specifically, the deficits in linguistic, visuospatial, and executive function were held to result from the disrupted connectivity between the posterior CB lobe (the medial and hemispheric regions of lobule VIIA Crus I/II, but also HVI and HVIIB) and cerebral association areas, especially prefrontal cortical areas in relation to executive control, parietal cortical areas with respect to visuospatial function, and frontotemporal regions in relation to linguistic function. Affective-emotional disturbance was seen as associated with lesions in the “limbic cerebellum,” associated with the vermis and fastigial nuclei connections with the reticular nuclei in the brainstem, intralaminar and anterior thalamic nuclei, the hypothalamus, as well as with the hippocampus, septum, amygdala, ventral tegmental area, periaqueductal gray and mammillary bodies, cingulate gyrus, and pregenual, retrosplenial, and paralimbic neocortical regions (for references, see [12, 14]).

The CCAS thus provided the concrete, clinical entity that lent support to the “dysmetria of thought” hypothesis and, more general, to a CB role in cognition and affect. As such, CCAS has been conceptualized as representing the third cornerstone of clinical ataxiology, the other two being the longer-established “cerebellar motor” and “vestibulo-cerebellar” syndromes [15].

As the question of relevance today no longer pertains to whether the CB plays a role in cognition and affect, but to the mechanisms by which this is accomplished [2], we hold that CCAS provides an ideal clinical entity for such an enterprise. This task force paper thus focuses on CCAS, reviewing its foundations, promoting awareness of its core components, tackling the skepticism articulated on its premises and relevant findings, discussing our current insight into its treatment, and identifying outstanding questions and future directions.

To this end, we have gathered contributions from experts in CB neurocognition. The section “Cerebellar Functional Topography in CCAS: Updates and Challenges” (Argyropoulos and Ivry) provides a critical introduction to CB functional topography, and the section “Cerebellar Neurocognition: Relevance to CCAS” (Stoodley and Van Overwalle) summarizes the neuroimaging evidence for a CB role in multiple cognitive domains in relation to CCAS. In the “Introducing Cognition into Cerebellar Rehabilitation: Facts and Hopes**”** section, Adamaszek, Masciullo, and Molinari address the practical issues pertaining to the multimodal deficits in CCAS and reflect on the current insight on therapeutic options. In the “Replication of CCAS” section, Schmahmann and Leggio review the replication of the CCAS over the past two decades in adults and children with inherited and acquired CB diseases. Finally, the “Discussion: Consensus on CCAS and Future Directions” section (Argyropoulos, van Dun, and Ivry) summarizes the points of convergence of the different sections and identifies outstanding questions that require further investigation.

## Cerebellar Functional Topography in CCAS: Updates and Challenges (G.P.D. Argyropoulos and R.B. Ivry)

### Introduction

Recognition of the impact of CB dysfunction across multiple task domains has accumulated over the last 30 years. Lesion–behavior relationships have been investigated with patient [16–18] and noninvasive brain stimulation studies [19]. Correlational evidence has come from neuroimaging studies, primarily involving either task-based fMRI [20–22] or resting-state functional connectivity [23–25]. Coupled with anatomical and physiological investigations in animal models [26, 27], these studies have helped develop a picture of CB functional topography, providing insight into the specific deficits in CCAS. In this section, we provide an overview of this topography and outline future research questions for CCAS.

### Basics of Cerebellar Functional Topography

CB functional topography is often seen in a quadripartite distinction of gross functional regions: the “vestibular,” “motor,” “cognitive,” and “limbic cerebellum” [10, 15]. Despite the considerable variation in the strength of the evidence, the literature indicates that distinct syndromes are associated with damage to these regions (e.g., [17, 28]).

#### The “Vestibular” and “Motor Cerebellum”

The vestibular CB comprises the flocculus-paraflocculus, the nodulus-ventral uvula (lobules IX and X^1^), and the oculomotor vermis (V–VII), with much of the output of these regions constituting the fastigial oculomotor region. The flocculo-nodular lobe (X) receives afferent projections from the vestibular nuclei. The vestibulo-CB syndrome is characterized by deficits of oculomotor movements, ocular misalignment, and instability [15].

Regarding the “motor cerebellum,” a broad range of studies, from physiological investigations in cats [31–33] to fMRI studies in humans [21, 34], have delineated at least two somatotopic CB representations [33, 35, 36]: a primary sensorimotor region (anterior lobe and adjacent VI) and a secondary region (lobule VIII). Tract-tracing in monkeys has established reciprocal connectivity between the primary motor cortex and lobules V, VI, VIIB, and VIII [1, 2] through feedforward corticopontine projections [37] and feedback projections via the interposed and dorsal dentate nuclei and thalamus [38]. Likewise, hemodynamic activity in human sensorimotor cortical regions correlates with that in the contralateral CB anterior lobe, the adjacent VI, and VIII [23–25, 39]. Hand, foot, and tongue movement activates the same lobules [21, 22, 40–44].

**Fig. 1:**
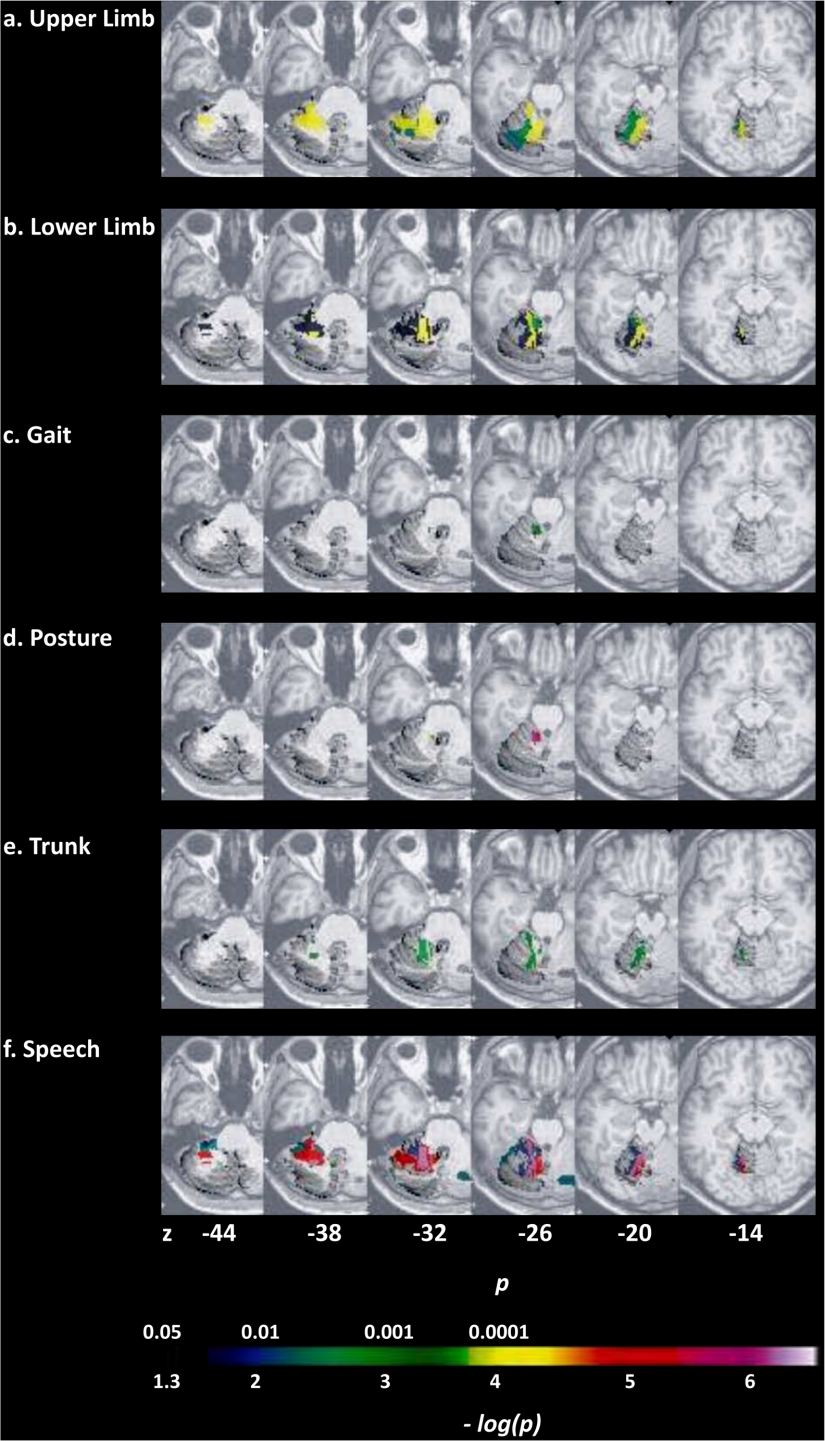
Highlights of advances in CB motor topography made by VLSM. **a**–**f** Lesion-symptom mapping analysis for subscores of the International Cooperative Ataxia Rating Scale (ICARS) [52] in patients with acute ischemia. **a** Upper limb ataxia correlated with lesions in vermal, paravermal, and hemispheric IV–VI. **b** Lower limb ataxia correlated with lesions in vermal, paravermal, and hemispheric III–VI. Limb ataxia correlated with lesions in the interposed and parts of the dentate nuclei; ataxia of gait (**c**), posture (**d**), and trunk (**e**) correlated with lesions in vermal and paravermal II–IV and lesions in the fastigial and interposed nuclei. **f** Dysarthria correlated with lesions in paravermal and hemispheric V–VI. Figures adapted from [45] © 2005, with permission from Elsevier

The CB motor syndrome is characterized by disequilibrium, ataxic gait, impaired limb coordination, and dysarthria. Upper limb ataxia is associated with lesions in the anterior lobe, adjacent regions of lobule VI, the interposed nuclei, and the dorsal dentate [17, 28, 42, 45–47]. Likewise, dysarthria is linked to damage in vermal VI (sensorimotor representation of the articulatory apparatus [48]), paravermal V–VI, and the dentate nucleus [49–51]. Detailed somatotopic evidence is shown in a large lesion-symptom^2^ mapping study [45] (Fig. 1). It remains unknown how damage restricted to posterior motor regions impacts movement. Damage there is less consistently associated with impaired motor learning as compared to anterior motor regions [53] and may not show lasting motor deficits [45].

#### The “Cognitive and Limbic” Cerebellum

Cognitive functions are associated with much of the posterior lobe (HVI, (H)VIIA Crus I/II, HVIIB, and (H)IX). In monkeys, polysynaptic pathways connect area 46 with Crus I/II and IX [38], and pontine input arises from frontopolar area 10 through to the most posterior regions of area 8 [8]. Moreover, the principal olive is linked with the lateral CB and the dentate nucleus (see[85, 281]). The likely anatomical substrate supporting a CB influence on autonomic/affective/limbic-related behavior is the connectivity of the vermis and the fastigial nucleus with subcortical limbic areas, associative and paralimbic cortical regions (see [27] for review). There is also evidence for anterior cingulate projections to medial Crus I and II [32]. Physiologically, vermal stimulation has been shown to modulate hippocampal, amygdala, and septum firing patterns [55].

Cortical-CB connectivity has been studied in humans with resting-state functional connectivity studies, investigating multiple CB networks, similar to those in the cerebral cortex. The networks in neo-CB regions are associated with prefrontal, posterior parietal, middle/superior temporal association cortex, as well as limbic and paralimbic regions [23–25, 39]. Nevertheless, there is little evidence to date to suggest a segregation of neo-CB regions in terms of functional connectivity with limbic versus association cortices.

In task-based fMRI studies, activations related to cognitive processes are typically observed in VI, Crus I/II, and IX. Similar regions are activated in contrasts designed to identify cognitive control processes [20, 21]. Studies of affective processing also disclose CB activation, albeit in both hemispheric and medial regions (HIV–HVI, HVIIA Crus I/II, X, vermal Crus II [20–22, 56]).

#### Relating Function to Structure in Cerebellar Disease

CCAS, by definition, postulates that impairments in cognitive and affective function are associated with CB damage. Much of this literature has involved the use of standard instruments to provide a neuropsychological profile in different patient populations (e.g., genetic subtypes, focal lesions, developmental abnormalities). In terms of cognition, the picture is somewhat contentious, with considerable variation across studies. In general, patients perform within normal bounds on perceptual and memory tests. Impairments, when observed, are on tests designed to assess executive function, similar to what is observed in patients with prefrontal lesions, although the impairments are generally milder [57]. The affective component of CCAS is assessed in a less systematized fashion, with many studies primarily employing clinical psychiatric assessment, with psychopathological diagnoses based on DSM [58, 59]. Our understanding of CB contributions to affect will be strengthened by more comprehensive employment of appropriate neuropsychological tests in larger patient samples (e.g., [13]).

Given the CB functional compartmentalization, patient data can be used to examine the correspondence between symptoms and lesion location. Much of this work has been based on case studies, but a few groups have undertaken larger-scale studies, employing more sophisticated statistical tools. Such investigations associate motor impairment with damage in the anterior lobe extending to VI and cognitive dysfunction with posterior CB damage (“Cerebellar Neurocognition: Relevance to CCAS” section).

**Fig. 2:**
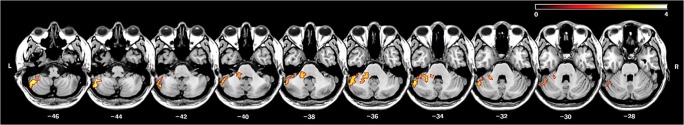
CB VLSM on depressive symptom severity. Figure adapted from [61] © 2017, with permission from Elsevier

The affective component of CCAS has been associated with damage in the posterior vermis and the fastigial nucleus [12, 58–60]. In the initial report of CCAS [12], the changes in affect in individuals with acquired CB lesions tended to resolve with time, suggesting that the affective changes may be due to remote disturbance in other regions. However, individuals with abnormal midline CB development show persistent changes in affect, but also cognitive impairment, even if the pathology is restricted to the vermis [58]. To date, there has been minimal sophisticated lesion-symptom mapping in the affective domain. One exception is a recent voxel-based study by Kim and colleagues [61], where left posterior CB damage was associated with depressive mood severity in 24 patients with isolated CB stroke (Fig. 2).

### Challenges and Future Directions

Considerable progress has been made in CB functional topography, which, coupled with the extensive neuropsychological literature, provides a firm foundation for understanding the pathology of CCAS and the symptom–lesion relationships of this syndrome.

#### Beyond Lobe- and Lobule-Based Lesion-Symptom Mapping

To date, the majority of lesion-symptom mapping studies have involved relatively crude grouping divisions: patients may be clustered as “vermal versus hemispheric,” “posterior versus anterior lobe,” or based on a simple lobular scheme [16, 28, 60, 62, 63]. Relatively few (e.g., [17, 45]) have used more sophisticated methods, such as voxel-based lesion-symptom mapping (VLSM) [64], in which MRI data are transformed onto standard atlases to allow inferential statistics based on voxel overlap, in part because these studies require relatively large samples. These approaches offer great promise for developing a refined picture of lesion-symptom mapping [65], one that can help shift the field away from its traditional lobe- or lobule-based perspective, incorporate the amplified sequelae of lesions in deep CB nuclei (e.g., [45]), and examine intra- and cross-lobular functional regions, since resting-state networks do not conform to lobular boundaries [24].

Structure–behavior mapping methods can be employed with diverse populations, including patients with degenerative disorders, stroke, and tumor. They offer a powerful tool to understand CB contributions to different developmental and psychiatric conditions, since CB hypoplasia is correlated with a number of those (e.g., autism, ADHD, schizophrenia, fragile X syndrome). Here, we have the opportunity to explore the specificity of impairments associated with early CB abnormalities, but also look at compensatory effects. Nevertheless, it will be important to not limit these studies to an analysis of CB dysfunction; in many conditions, pathology extends into the brainstem, diencephalon, and cerebral cortex. To date, we are not aware of any studies that have asked how the behavioral consequences of damage to a particular CB region are modulated by correlated pathology in extra-CB structures.

#### Beyond the Cerebellum

This last point is also relevant when considering CCAS, even in focal CB pathology. One feature of the original paper linking affective disturbances to CB pathology is that the symptoms were transient in many patients [12]. Subsequent work has shown that affective changes can be chronic, observed in patients with spinocerebellar ataxias (SCAs) [66] or adult survivors of childhood CB insults [63]. Understanding the time course of different clusters of symptoms can provide important clues concerning whether symptoms arise directly from disruption of CB processing or indirectly from off-target effects [67].

More generally, a network-based approach is essential for understanding CB function across domains. CCAS involves deficits similar to those following lesions in the cerebral nodes of the corresponding cerebro-CB loops [12]. Likewise, recent work suggests that deficits traditionally associated with basal ganglia (BG) pathology such as dystonia may actually reflect disruption of CB–BG interactions [68], a point underscored by evidence of reciprocal, multimodular CB–BG connectivity [69–71]. Future research should examine CB contributions on cognitive processes traditionally associated with the BG, such as probabilistic learning or reinforcement. Not only will this work help sharpen our description of CCAS, but the study of patients with acute lesions can be used to ask if CB pathology disrupts BG function.

A network perspective is also warranted to reconsider memory function and the CB, in light of the default-mode network hubs in posterior Crus I/II and IX [24]. To date, hippocampal–CB connectivity [72–75, 282] is primarily discussed with respect to a CB role in affect [12, 27], but not in relation to prominent hippocampal functions, i.e., episodic memory and navigation [76], despite the evidence supporting CB involvement in navigation [77, 78] and episodic memory [79, 80]. Interestingly, a meta-analytic study on neurodegenerative conditions disclosed CB atrophy in Alzheimer’s disease (right HVI/HVIIA Crus I/II [81]), which was discussed in relation to CB–hippocampal functional connectivity [82]. Here, too, would be an opportunity to explore cognitive deficits in Alzheimer’s disease as a function of the extent of CB pathology.

#### Beyond Heuristic Labels

Finally, it is important to recognize that functional labels (“cognitive,” “language,” “executive,” “affect”) are just heuristics and that applying such labels may impede our understanding of computational principles. For instance, anatomical, functional, and symptom-based analyses converge on the idea of two CB motor zones: an anterior one (lobules I–VI) and a more posterior one (lobule VIII). While the role of the latter deserves further investigation, data also implicate damage to lobule IX in upper limb dysfunction [17, 83]. This region may be essential for the visual guidance of movement [84], given its connections with visual association areas [85]. Thus, we may need to revise our methods for differentiating “motor” from “cognitive,” open to the idea that CB function may require considering an interface between higher-level functions and motor-like operations. For example, the internalization of speech mechanisms in covert rehearsal is an important part of verbal working memory [86] (“Cerebellar Neurocognition: Relevance to CCAS” section).

### Conclusion

We provided an overview of CB functional topography in relation to CCAS, highlighting some of the gaps in this literature, in terms of CB regions and their interactions with extra-CB structures. Our understanding of CCAS and CB function will benefit from more rigorous work using VLSM. Here is an ideal situation to develop multisite collaborations, since the sensitivity of these studies is greatly improved with large samples.

## Cerebellar Neurocognition: Relevance to CCAS (C. Stoodley and F. Van Overwalle)

### Introduction

Data from multiple sources support a role for the human CB in cognitive function. Understanding the specific CB regions that support cognition and the CB contribution to cognitive functions are relevant to our understanding of the CCAS. Drawing upon evidence from healthy populations and studies of CB patients, we summarize the neuroimaging evidence that the CB supports cognition in multiple domains, the relevance of such findings to understanding cognitive deficits in CB patients, and the theoretical constructs of what the CB might contribute to cognitive function.

### Cerebellum and Cognition: Evidence from Neuroimaging

**Fig. 3:**
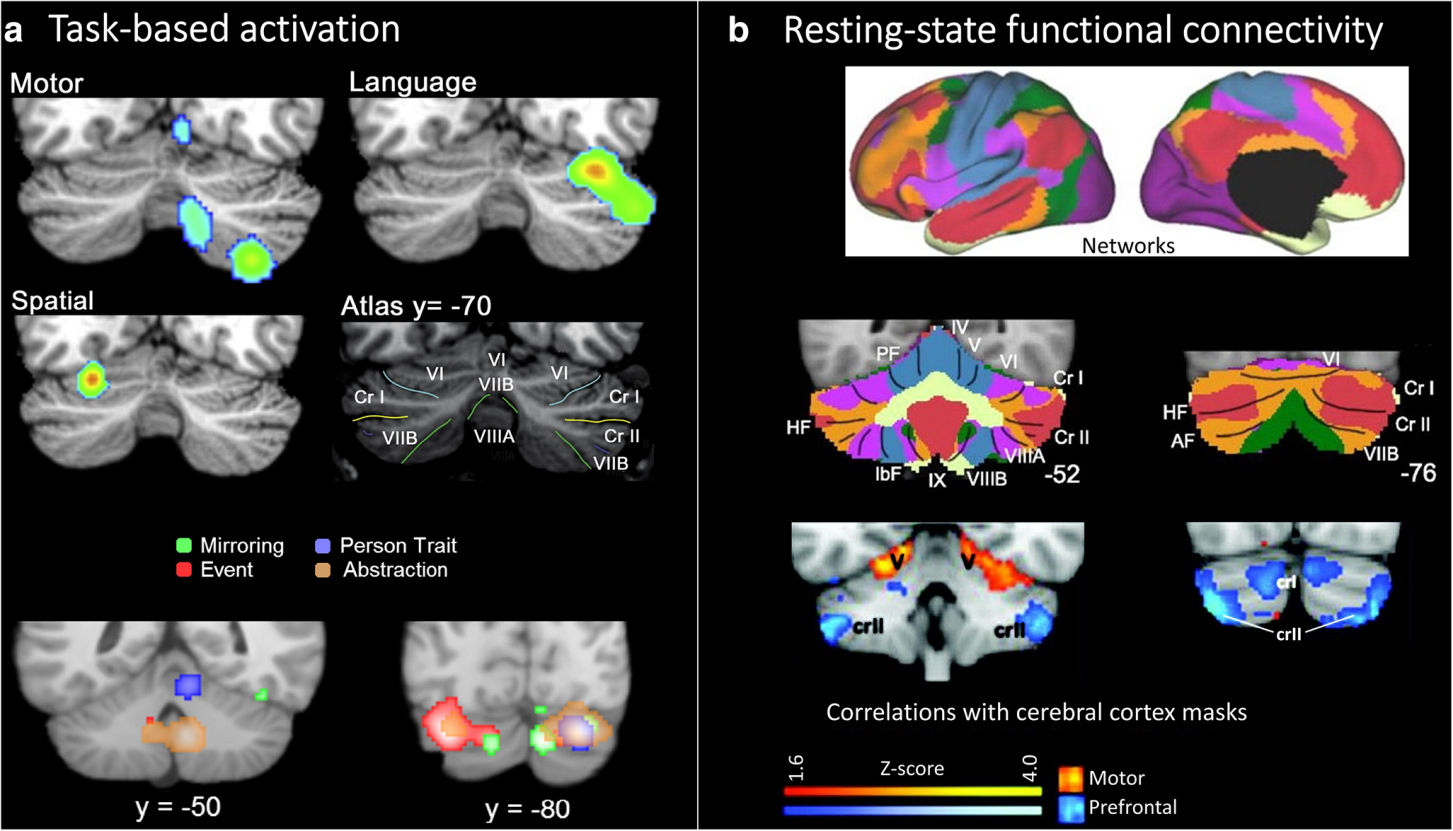
CB engagement in cognitive tasks and cerebro-CB networks supporting cognition. **a** (Top) A meta-analysis of task-based activation patterns reveals CB activation during language and spatial tasks differs from CB regions engaged during motor tasks (modified from [21], with permission). (Bottom) A meta-analysis of task-based activation patterns reveals CB activation during social mirror-related tasks (mirroring) and social mentalizing-related tasks (inferring intentions behind events, personality traits, and more abstract inferences including a person’s past and future) (modified from [88],with permission). **b** (Top) Resting-state functional connectivity shows that the CB is part of resting-state networks supporting cognition, including the frontoparietal control network (orange) and dorsal (green) and ventral (violet) attention networks (modified from [24], with permission). (Bottom) A similar pattern is seen when CB functional connectivity with motor (orange) and prefrontal (blue) masks are used (modified from [25], with permission)

Neuroimaging studies in healthy individuals consistently report CB activation during a wide range of cognitive tasks (for reviews, see [20, 21, 87]), consistent with the idea that the CB is part of a network of regions supporting cognitive function. Resting-state fMRI studies demonstrate CB functional connectivity with cerebral cortical regions involved in cognitive processes (e.g., the prefrontal cortex [25]), and more broadly with the frontoparietal, dorsal/ventral attention, and mentalizing/default networks (e.g., [24]). Within the CB, there is a broad distinction between regions that are engaged during sensorimotor tasks and show functional connectivity with primary motor and somatosensory cortices (anterior lobe extending into medial lobule VI and lobule VIII) and regions showing activation during cognitive tasks and functional connectivity with frontal and parietal regions (posterolateral CB; Fig. 3). Neuromodulation of the posterolateral CB has been shown to impact functional connectivity with prefrontal and parietal networks, with no effect on functional connectivity with sensorimotor regions (e.g., [89, 90]), which is consistent with the anatomical connections between the CB and cerebral cortices (see [26, 91]).

The CB is active during functional localizers for language and is considered part of language networks ([92]; see [93] for a consensus paper). A variety of language and reading tasks, including verbal fluency, verb generation, and sentence completion, engage the CB (see [87] for review), and CB neuromodulation impacts performance on similar paradigms (e.g., [94–96]). The predominantly left-lateralized cerebral cortical activation during language paradigms is mirrored by right-lateralized posterolateral CB activation, reflecting the contralateral connectivity between the CB and cerebral cortex (see [97]). Notably, anterior and medial CB regions are engaged during articulation, whereas cognitive linguistic task activation is more posterior and lateral [97], reinforcing the idea that CB contributions to cognition are not contingent on overt motor control.

Working memory, spatial, and executive function tasks also engage CB circuits. Working memory paradigms, such as the n-back and Sternberg tasks, activate lobule VII bilaterally and right VIII; spatial tasks, including mental rotation and line bisection, engage bilateral lobule VI; and executive function paradigms, such as the Wisconsin Card Sorting Task, also involve bilateral CB regions, predominantly lobule VII (see [20, 21]). Meta-analyses show that there are both distinct and overlapping CB regions involved across these tasks [20, 21], depending on task demands. For example, within subjects, activation associated with verbal working memory overlaps in right lobule VII with activation during covert verb generation (e.g., [43]), reflecting shared linguistic components of these tasks. In a large sample from the Human Connectome Project, a recent study investigated CB activation patterns for working memory, language, social processing, and emotion processing [22] and showed largely distinct activation patterns associated with different cognitive measures and revealed cognitive task activation in lobules IX/X in addition to VI and VII.

More recently, the CB role in social cognition has gained increasing recognition. A meta-analysis ([88, 98]) showed that about one third of all studies on social cognition engaged the CB when the tasks involved social mirroring (e.g., observing others’ intentional body movements) or mentalizing (e.g., inferring others’ intentions, beliefs, and personality traits on the basis of behavioral descriptions; Fig. 3). Roughly, the mirror versus mentalizing tasks follow the same anterior sensorimotor versus posterior nonmotor distinction in the CB. Functional connectivity analyses on social cognition confirmed task-related connectivity between the anterior CB and activation in mirror cortical areas, while the posterior CB (mainly Crus I) showed task-related connectivity with cortical areas involved in mentalizing [99, 100]. Further, a recent repetitive transcranial magnetic stimulation (rTMS) study found that CB rTMS interfered with implicit social biases [101].

### Relationship to CCAS: Do Patient Outcomes Reflect Imaging Patterns Seen in Healthy Individuals?

**Fig. 4:**
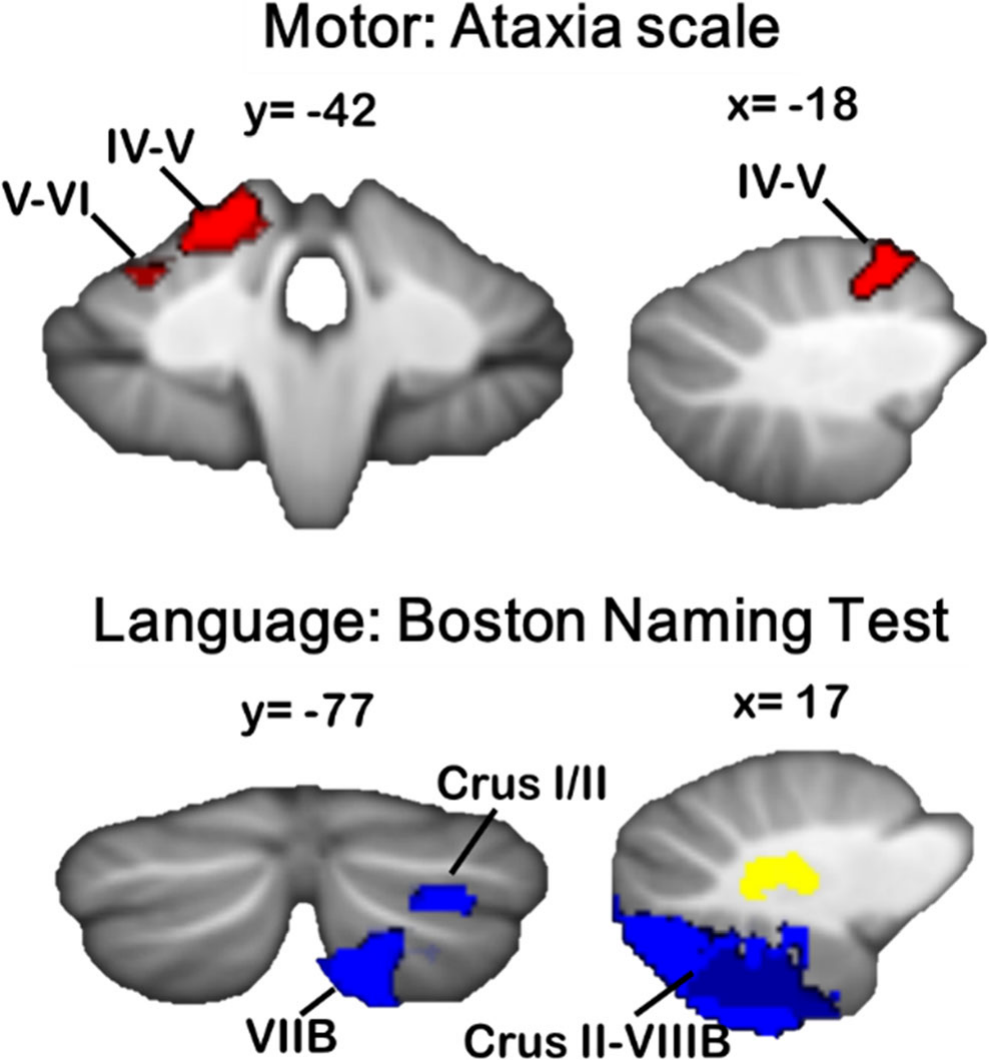
VLSM reveals CB regions associated with cognitive versus motor deficits following CB stroke. Significantly poorer ataxia symptoms were associated with damage to the anterior CB and lobule VI (top), whereas poorer performance on the Boston Naming Test was associated with right-lateralized damage to posterior CB regions, including Crus II, VIIB, and VIII (bottom). Adapted with permission from [17]

Findings from neuroimaging studies in healthy individuals suggest that cognitive deficits should result from damage or degeneration involving posterior CB regions. Indeed, in its very first description, it was noted that the CCAS tends to result from lesions affecting the posterior CB lobe [12]. Evidence from pediatric CB damage and developmental disorders also suggests that the anterior “motor” versus posterior “cognitive” dichotomy is present early in development and predicts later outcomes (for review, see [102]). VLSM in CB stroke patients showed that the CB motor syndrome was associated with anterior CB lesions, whereas CCAS resulted from posterior CB damage [17]. Consistent with task-based functional imaging, worse motor symptoms (pegboard, tapping, ataxia scores) resulted from lesions to the anterior lobe, whereas impaired language performance (e.g., Boston Naming Test) was associated with right-lateralized damage to lobule VII (Fig. 4). Among the cognitive tasks, there was also variation in the CB regions where damage resulted in poorer task performance; for example, performance on the Wisconsin Card Sorting Task was associated with damage to lobules VII and VIII, whereas poorer performance on Trails A and B was associated with lesions involving lobules IV–VI. These findings are consistent with neuroimaging activation patterns in healthy individuals.

SCA patients also show an association between the posterior lobe and cognitive performance. Kansal and colleagues reported that cognitive scores in CB degeneration (e.g., verb and phonemic fluency, working memory, cognitive flexibility) were associated with the volume of posterior lobe regions, including lobules VI, VII (Crus I, Crus II, and VIIB), and IX [80]. In aging populations, similar relationships were seen between posterior CB volumes (e.g., Crus II, VIIB) and cognitive function [103]. Functional connectivity analyses support these structural imaging findings: in SCA2, lobules VI and VII showed reduced functional connectivity with cortical regions supporting cognition and emotion, including the superior and middle frontal gyri [104]. Such changes in cerebro-CB network connectivity have also been associated with clinical impairments in visual–spatial processing and executive function in SCA6 [105].

In social/emotional cognition, patients with CB disorders show impairments in attributing facial expression to the correct emotional or mental state [106–109]; for a review, see [110]). With respect to understanding social behavior, several studies reported that CB patients have impairments in identifying or generating a plausible sequence of pictures reflecting a complex action performed by a human agent [111, 112]. Patients were particularly impaired when correct sequencing required mentalizing about the agents’ beliefs, but not so for routine social scripts [113]. Likewise, Zalla and colleagues [114, 115] found that children with autism spectrum disorder (ASD) were impaired in predicting the outcome or sequence of human behavior, but were less impaired in understanding routine social interactions. Indeed, there are strong associations between CB dysfunction and ASD (for reviews, see [116–118]). Studies have reported altered connectivity between the CB and cortical areas in ASD [119, 120], and CB gray matter volume correlates with the degree of social impairment in ASD (e.g., [121]).

As both functional neuroimaging and lesion-mapping studies increase in number, CB regions associated with more specific aspects of cognition will likely emerge, leading to improved prediction of cognitive outcomes in CB patients.

### Which Aspects of Cognition Does the Cerebellum Support?

It has been proposed that the relatively uniform CB circuitry supports a common processing mechanism (e.g., [26, 122, 123]), the UCT [3]. The CB is thought to build motor or mental internal models [124], which are trained based on error signals and used to predict the consequences of ongoing motor or mental processes [122]. This enables the CB to participate in processes important to optimal cognitive function, including prediction [125] and performance monitoring [126]. In cognition, prediction has most often been studied in the context of language (see reviews in [127, 128]). For example, CB neuromodulation disrupted performance when the first part of a sentence generated a strong prediction, but not when sentences did not have a strong predictive context [95, 96]. Neuroimaging studies have shown CB activation associated with linguistic predictions [90, 129, 130] and violations of those predictions [129, 130] during sentence processing. However, imaging or stimulation studies that explore the mechanisms underlying impaired social action sequencing and their implications are lacking.

Now that the CCAS has been described in multiple CB patient populations and its neural correlates are starting to be delineated, the field can focus on establishing a better understanding of which aspects of cognition are impaired in CB disease. This will entail designing measures that tap specific features of cognitive processes (e.g., error monitoring, prediction, sequencing) to determine whether a common mechanism can be identified that, when damaged, leads to the deficits seen in CB patients.

### Conclusion

Evidence from neuroimaging and patient populations suggests that the posterolateral CB contributes to cognitive processing via anatomical connections with supratentorial regions supporting cognitive function. Future investigations should aim to clarify the effects of regional CB damage on specific aspects of cognition, and to determine the CB contribution to these functions. This information will improve the ability to predict which CB patients may be diagnosed with the CCAS, and provide targets for remediation of cognitive deficits in CB populations.

## Introducing Cognition into Cerebellar Rehabilitation: Facts and Hopes (M. Adamaszek, M. Masciullo, and M. Molinari)

CB damage has been associated with several movement disorders including incoordination, reduced manual dexterity, postural instability, and gait disturbances [131–133]. Patients with CB lesions obviously should participate in conventional rehabilitation interventions, including speech therapy, coordination exercises, therapy for balance, mobilization, motor re-education, as well as various occupational therapy activities [134].

Historically, in line with the classical view of the CB as a motor center, the main focus of rehabilitation approaches has been on motor aspects, mainly ataxia and dysmetria [135, 136], although there is limited evidence about their effectiveness [137–140].

Recovery following CB damage is, indeed, slow and often incomplete [141], and it has been suggested that individuals with focal lesions have better recovery than those with diffuse lesions [134, 142]. Moreover, as the result of the modulatory CB role upon remote structures (such as the cerebral cortex), CB injury may give rise to a constellation of behavioral, affective, and cognitive symptoms (CCAS), that may further impact function and the rehabilitation process.

Indeed, regarding function recovery, Ilg and colleagues proposed that, after CB damage, walking is no longer automatic, and as every step would be a conscious movement, pathways through the (“cognitive”) cerebro-CB would be engaged [133]. Therefore, if there is damage throughout the CB, individuals would not only experience the difficulties resulting from the damaged (“motor”) spino-CB but would also have difficulties compensating due to the cerebro-CB damage.

The key CB role in motor learning and adaptation following extra-CB pathology [135, 143] may limit functional recovery in people with CB dysfunction. Appreciating the central role of the CB in motor learning and learning through error informs our understanding of why individuals with CB lesions take longer to relearn the skills of walking and require more repetition than individuals with other central nervous system lesions. In this context, it may also be necessary to use compensatory aids and strategies for those with more severe CB damage.

As previously mentioned, CB lesions may cause loss of activity within the cerebral cortex due to the large interconnections between these structures. In this context, there has been significant advances in the CB neuroscience of cognitive functions, mainly provided by conceded clinical and particularly neuroimaging protocols. High-resolution structural and functional MRI and recent developments in fiber-tracking techniques such as DTI and DSI yielded deeper anatomical insight to the CB and its connections to incoming and forwarding signal connections to cerebral areas, among them the prefrontal, parietal, and temporal cortices in accordance with cognitive domains of attention, working memory, and a broad range of executive functions, including cognitive– and affective–behavioral control [21, 144]. Moreover, CB rTMS has started to disentangle the widespread scale of the underlying neurophysiological signal patterns of different CB network connections to the cerebrum [145]. Finally, yet with less clinical impact, EEG recordings implementing MEG and ERP protocols [146] have been proven to enrich principal research in the cognitive CB. This is in order to delineate the time course and, therefore, the temporal order in which CB areas are part of the network activities of attention and cognitive– and affective–behavioral control. These technologies of studying CB pathways in healthy individuals and patients seem to be not only of ongoing interest to forward the current scientific development, but mature for application in clinical requests. Indeed, they allow the identification of the causal CB contribution to cognitive impairment and, furthermore, are useful for guiding the indicated neurorehabilitative therapy most appropriate.

Resolution of cerebro-CB diaschisis after CB lesions may also underlie symptom recovery [147, 148]. In humans with CB dysfunction, for example, an increase in the activation of the medial premotor system while moving has been reported, which may feature a compensation for the lack of activation of the lateral premotor areas that receive extensive CB input [149]. Torriero and colleagues hypothesized that isolated left CB damage may reduce excitatory drive to the contralateral right dorsolateral prefrontal cortex, resulting in an imbalance in activity between the left and right cortex. Readdressing this imbalance temporarily by inactivating the left dorsolateral prefrontal cortex (rTMS) resulted in improved procedural learning [150]. Considering the emerging demand for neurorehabilitation of cognitive impairment due to CB disorders at a clinical neuropsychological level, a growing spectrum of increasingly powerful computer-based intervention programs, personal teaching methods (e.g., metacognitive or cognitive–behavioral training) including an individual reward option and the frequent task to engage patients’ awareness of afflicted cognitive facilities, and external support by time-scheduling devices (e.g., a pager), are feasible recommendations [151]. Proof-of-principle protocols of transcranial stimulation (tDCS, rTMS) are of growing interest, at least for enhancing the clinical neurotherapeutic approaches. These require a strong investigational consideration in clearly described therapeutic protocols regarding some crucial issues, such as the etiology of CB disorder (focal vs. extensive degenerative), the type and site of stimulation, but also the cognitive domain of interest [152]. Similarly, confined physiotherapeutic and occupational therapeutic applications could potentiate the clinical outcome in cognitive disabilities in CB disease, especially if considered in corroborating motor rehabilitation intention. Neuropharmacological aspects of supporting the endeavors of CB–cerebral function restoration as in particular provided along serotonergic and GABAergic receptor profiles might be of interest in outstanding clinical studies. Notwithstanding, an individual but sophisticated combination of clinical and neurophysiological diagnostic protocols, the latter implementing fMRI, rTMS, and/or ERP paradigms to depict morphometric and/or temporal indications of therapeutic-dependent changes in cognitive functioning, is recommended to evaluate the effectiveness of multimodal cognitive interventions in order to advance the quality of outcome measurement [153].

Given the central CB role in cognition, patients with CB lesions should be assessed for cognitive deficits in the course of their overall evaluation during rehabilitation. The data obtained from these assessments will be helpful for treatment planning. This may include (i) understanding memory impairments that limit patients’ ability to retain elements of the treatment regimen, (ii) determining difficulties with comprehension and abstract reasoning that could interfere with the ability to process treatment information and to function safely upon discharge, and (iii) identifying visual–perceptual–motor deficits that could compromise patients’ acquisition and usage of important environmental information and cues (and could further risk safety).

The individuation of specific cognitive profile should be, therefore, mandatory in cognitive rehabilitation of patients with CB signs. Although CB-induced deficits are typically more subtle in adults, the clinical symptoms clearly resemble those caused by supratentorial lesions [12, 15]. Nevertheless, evidence suggests that the CB-induced cognitive deficits should be treated in a manner different from similar cognitive deficits arising from cortical damage [154]. Although systematic studies are clearly warranted, available evidence suggests that CCAS should be treated in a specific way. Approaches where the patients are explicitly made aware of their deficits and are assumed to act as an “external cerebellum” could be considered the most promising in the future [54].

Moreover, a question which requires further research is the elucidation of the patterns of recovery following an acute CB lesion versus a more complex impairment of the CB–cerebral pathways. For the motor CB syndrome, a stage-by-stage recovery process has been uncovered [155]. Regarding CCAS, we are facing a gap in understanding how recovery evolves. Whether the motor deficits and the cognitive/affective deficits are correlated remains unclear. Further research in embodiment mechanisms is of high interest for understanding the specific functional organization of CB–cerebral networks in coupling motor and nonmotor sequences.

## Replication of CCAS (J. D. Schmahmann and M. Leggio)

### Introduction

The emerging field of CB cognitive neuroscience coalesced with the description of CCAS first in adults [12] and then in children [60]. It underscored the clinical relevance of the new field of the CB cognitive neuroscience and had immediate implications for the diagnosis and care of patients with neurological and neuropsychiatric disorders. It also demonstrated the clinical relevance of the rediscovery of early suggestions about a CB role outside motor control, the reinterpretation of early anatomical studies, novel anatomical observations of CB incorporation into the distributed neural circuits subserving a wide range of human behaviors, early functional imaging studies showing CB activation in cognitive tasks, and new theories about CB function [156].

When Manto and Mariën [15] introduced the eponymous designation “Schmahmann syndrome” for the CCAS, they conceptualized it as the third cornerstone of ataxiology, along with the motor and vestibular disorders. Converging data now reveal that the sensorimotor CB has a primary representation in the anterior lobe bordering on lobule VI and a second representation in lobule VIII; and the cognitive–limbic CB has three representations in the posterior lobe—lobule VI/Crus I, Crus II/VIIB, and lobules IX/X [21, 22, 24]. The high degree of functional topography in these CB cortical areas parallels the connectional specificity in anatomical studies [157] and the intradomain cognitive topography within individual lobules (e.g., different aspects of working memory recruit adjacent regions of lobule VIIB; [158]). It has been proposed that the CCAS represents the clinical manifestation of dysmetria of thought, the result of disruption of the UCT applied to the loops of information processing that subserve cognition and emotion in addition to sensorimotor control [3, 5]. It therefore has broad ramifications for understanding the mechanisms of cognition because it necessitates the incorporation of the CB and other subcortical nodes into the neural circuits relevant to human behavior [157, 159, 160].

Here, we review the replication of the CCAS over the past two decades in adults and children with inherited and acquired CB diseases. For comprehensive reviews, see [2, 3, 10, 11, 13, 54, 93, 156, 157, 161–167].

### Cerebellar Stroke

Lesion-deficit studies in patients with focal injury provide pivotal insights into structure–function correlations. When stroke involves the superior CB artery (SC-Art) which irrigates the anterior lobe and adjacent sectors of lobule VI, the clinical features conform to the CB motor syndrome of gait ataxia, ipsilateral limb dysmetria, and dysarthria [48, 51, 168–173]. VLSM demonstrates somatotopic representation of the limbs, trunk, gait, and speech [45]. The SC-Art territory is not exclusively confined to the anterior lobe, and visual–spatial deficits have also been described following SC-Art stroke [174].

Functional topography in the CCAS was evident from the outset, occurring in patients with posterior lobe stroke [12]. In 39 subsequent CB stroke patients [28], 26 (66.6%) had the CB motor syndrome, but 13 (33%) were motorically normal. Motor findings were in patients with anterior lobe lesions, whereas those with stroke in lobules VII–X had no ataxia. A VLSM study [17] confirmed and extended these observations with a double dissociation: stroke in the anterior lobe produced the CB motor syndrome but not the CCAS, whereas stroke in the posterior lobe produced the CCAS but not the motor syndrome. These observations are consistent with the CB functional topography in healthy controls with task-based [21, 43, 175] and resting-state fMRI [22, 24].

Review of the CB stroke literature reveals that all CCAS elements may occur following focal CB lesions. Some of these reports are highlighted here.

- Posterior inferior CB artery (PICA) territory strokes in the right CB hemisphere degraded error detection, practice-related learning of a verb-for-noun generation task [176] and produced agrammatic speech [177].
- Eighteen young adults with isolated CB stroke were impaired on tasks of working memory, motor speed, and integration of visual, spatial, and motor skills [178].
- Fifteen patients with isolated CB infarcts (PICA, 10; SC-Art, 4; AICA 1) had executive dysfunction with impaired phonemic and alternating category fluency, naming with and without interference, and paced auditory serial addition task, visual–spatial deficits on the WAIS-R Block Design test, and personality changes including disinhibition [179].
- Six PICA stroke patients had deficits in visual–spatial working memory, attention and verbal episodic memory, and elevated scores on psychopathology scales [180].
- Thirty-seven patients with isolated CB infarcts had elevated scores on a frontal systems impairment index, delayed recall of verbal or visual information, anomic aphasia, limb kinetic apraxia, and acquired dyslexia [181]. Another 43 with isolated CB or brainstem infarcts were impaired on tests of apathy, disinhibition, executive function, and emotional intelligence [182].
- Twenty-two (88%) of 25 Russian patients with isolated CB infarcts showed impaired attention, cognitive control, and mental flexibility [183]. Six (24%) with right PICA strokes had linguistic difficulties—naming, irregularity of speech, agrammatism, and aprosodia, and memory impairment with loss of previously acquired habits, facial agnosia, amusia, and temporal disorientation.
- Twenty-six patients with isolated CB infarcts demonstrated impaired working memory and visuospatial and visuomotor abilities, with greater deficits following right-sided lesions [184].
- In 19 patients with isolated CB lesions and 6 with idiopathic CB ataxia, verbal fluency was impaired in a modality-specific manner, phonemic fluency more impaired than semantic fluency [185]. Both left- and right-sided damage caused reduced verbal fluency, with a slightly greater right-sided predominance. Patients with CB degeneration were subsequently shown to produce fewer words than healthy controls, even when controlling for slowed articulation, with a trend for patients to produce fewer responses during the phonemic compared to the semantic condition [186].
- In a VLSM study of 21 adults with remote CB stroke (46.7 ± 17.0 months), impaired phonemic fluency correlated with lesions in the cortex and white matter of right Crus II, the deep nuclei, and lobules IX and X [187].
- The right CB was also implicated in decreased phonemic fluency in a study of chronic CB hemisphere lesions from stroke or tumor resection ([188], *n* = 22; [189], *n* = 32).
- Executive function, mental flexibility, focused attention, and real-life errand tasks were impaired in patients with CB injury [190] (*n* = 11), together with deficient reverse digit span [191] (*n* = 15), and impaired verbal working memory [192] (*n* = 9). Both components of verbal short-term memory are affected by CB lesions (the rehearsal system [193] and the phonological short-term store [194]), suggesting that the CB serves as an interface between the phonological short-term store and articulatory rehearsal, comparing the output of subvocal articulation with the contents of the phonological store [41].
- The essential elements of CCAS—language, executive function, and visuospatial abilities, were confirmed in a retrospective study of 156 patients, 78 with isolated CB lesions and 78 with CB atrophy [16]. Cognitive deficits were most marked in patients with lesions in the PICA territory and the deep CB nuclei. Further, sequencing deficits were the most marked cognitive impairment in all patients, with the exception of those in whom the CB nuclei were spared.

Case reports of CB stroke provide granular detail about the personal impact of the CCAS. These include emotional dyscontrol and aggression [195]; impaired language processing with decreased verbal fluency and semantic deficits [196–198]; transcortical sensory aphasia with impaired reading and writing [199]; spatial dysgraphia [200]; impaired verbal learning and memory [195, 201]; executive dysfunction including difficulty following complex conversations, making decisions, planning, abstract reasoning, set-shifting, and perseveration [196–199, 201]; loss of emotions [201]; psychomotor agitation, spatial–temporal confusion, alteration of personality with dysphoria, disinhibition, affective indifference to family, and panic disorder [197]; disinhibition with disorders of judgment and reasoning [202]; and cognitive slowing affecting visuospatial analysis [199, 202, 203].

### CCAS in the Hereditary Ataxias

Cognition is involved to varying degrees in the genetically defined SCAs [204]. These deficits conform to the pattern of the CCAS—deficits in executive function, linguistic processing, spatial cognition, verbal and visual memory, and changes in affect. Even SCA6, the purest CB form of the SCAs, has problems with executive function, and in the study of Hoche and colleagues [13], there were no group differences in cognition between patients with complex cerebro-CB disease versus isolated CB pathology except for a test of similarities in which complex patients were more impaired. Orsi and colleagues [205] observed memory, language, visuospatial, attentional, executive, and mood changes consistent with the CCAS in patients with SCA1, 2, 6, and 8, with no significant differences between subgroups. Cognitive changes deepen as the disease evolves, as described in SCA3 [206]. True amnestic dementia sets in late in the illness in some SCAs, likely reflecting neuropathology involving medial temporal lobe structures, because long-term recall is usually relatively spared in the CCAS [12, 13, 60], even though access to stored events and facts through executive control of recall is impaired. Lindsay and Storey [207] point out that disorders like SCA17 with disseminated neuropathology may produce widespread cognitive impairment including dementia.

CCAS is reported in hereditary ataxias in which neuropathology is thought to be restricted to the CB and its connections. Ataxia telangiectasia, a childhood-onset disorder from mutations in the ATM gene causing progressive CB degeneration [208], is associated with cognitive impairments that become more appreciable as the disease evolves [209, 210]. Autosomal recessive CB ataxia type 1, also confined to CB, has findings consistent with the CCAS [211]. And Friedreich’s ataxia, a predominantly afferent ataxia with involvement also of the deep CB nuclei, involves slowing of cognition [212] and impairments in verbal fluency, working memory, and social cognition [213].

Neuroimaging in the hereditary ataxias reveals that atrophy in different CB subregions may account for the specificity of cognitive symptoms. In patients with SCA2, atrophy in the cognitive CB in the posterior lobe (lobules VI, Crus I, Crus II, VIIB, and IX) correlated with impaired visuospatial, verbal memory, and executive function, whereas atrophy in the motor CB (lobule V in the anterior lobe; lobules VIIIA and VIIIB of the posterior lobe) correlated with motor deficits and impaired motor planning [214]. CB atrophy was also associated with altered diffusivity of the middle and superior CB peduncles, the main cerebro-CB afferent and efferent white matter tracts, respectively, indicating that cerebro-CB dysregulation may account for the CCAS in SCA2 [215]. Network-based statistics reveals that altered internodal connectivity between the CB posterior lobe and the cerebral cortex correlated with assessments of cognition and emotion, consistent with the view that CB dysfunction in SCA2 affects cerebral regions at a distance and that the clinical symptoms may be related to connectivity changes in both the cerebral and CB nodes of motor and nonmotor cerebro-CB circuits [104]. These findings are consistent with the observations that there are distinct and topographically precise CB contributions to cerebral intrinsic connectivity networks [24, 25, 39] and that rTMS applied to distinct CB regions can selectively modulate network functional connectivity in healthy individuals [145, 216]. Further, dysfunctional connectivity between the CB and the dorsolateral prefrontal cortex is associated with negative symptom severity in schizophrenia, and improvement of the connectivity ameliorates the severity of the negative symptoms [217].

### Language in the CCAS

Language difficulties in the CCAS included dysprosodia, agrammatism, anomia, impaired syntax, and deficits in verbal fluency and telegraphic speech [12, 177], while children with CCAS experienced expressive language deficits, word finding difficulties, and mutism in those with vermal damage [60]. Subsequent studies showed CB contributions to speech and language perception, grammar, motor speech planning, syntax processing, and the dynamics of language production including writing and reading skill [93, 200, 218]. CB patients are impaired on a word stem completion task [186] and on metalinguistics, the higher-level language function essential for social interaction, including the ability to engage with and respond to the contextual and situational demands of normal discourse [219, 220].

### Attention in the CCAS

Behavioral and neuroimaging studies demonstrate a CB role in attention, which is impaired in patients with the CCAS [14]. This topic has remained controversial [221]. In a study of SCA2 patients [222], Go/NoGo and divided and sustained attention were impaired. These tasks depend on multisensory integration, sequencing, prediction of events, and inhibition of inappropriate responses, all of which are affected by CB damage. Further, divided and sustained attention correlated with lobules VIIB/VIIIA which have been proposed to be part of the dorsal attention network [104, 223], and selective attention correlated with vermis lobule VI. These findings provide support for the involvement of specific CB regions in the attention impairments seen in the CCAS and for the inclusion of the CB within the dorsal attention network.

### Cognitive Sequencing in the CCAS

Sequence detection has been proposed as the operational mode of the CB in different domains [112, 164, 224–226]. Sequencing abilities are impaired in CB patients [16], for both sensory-motor [227–233] and cognitive domains [224]. Further, left-sided lesions impair the ability to detect and correctly reproduce sequences based on pictorial material, whereas right-sided lesions degrade sequencing ability when verbal elaboration is required [112].

### CCAS in Children

The CCAS occurs in children with CB stroke. Five boys (age 3–14) had mood disturbances, outbursts of laughter and/or crying, and alternating agitation or prostration. Cognitive deficits included mutism followed by anomia and impaired comprehension, planning, visual–spatial organization, and attention. The cognitive difficulties improved slowly and incompletely and were more disabling than the motor symptoms [234].

Central nervous system tumors disproportionately affect posterior fossa structures in childhood. Levisohn and colleagues [60] first addressed the question of cognitive change in children who underwent CB tumor resection without confounding brain radiation and use of methotrexate. In 19 children (age 3–14), they noted problems with attention and executive impairments in sequencing, planning, and establishing and maintaining set. Expressive language was characterized by reluctance to engage in conversation, long response latencies, brief responses, lack of elaboration, and difficulties with language initiation, word finding, and confrontation naming. Many had problems solving strategies, visual–spatial deficits, impaired verbal recall, and failure to organize verbal or visual–spatial material for encoding that impacted retrieval. Impaired regulation of affect following vermal damage manifested as irritability, impulsivity, disinhibition, and lability, with poor attentional and behavioral modulation. Subsequent studies revealed impaired executive functions with deficient planning, sequencing, mental flexibility and hypothesis generation and testing, also involving visual–spatial function, expressive language, and verbal memory [235–241]. Similar phenomena were observed by Riva and Giorgi [59] (*n* = 26), who reported impaired verbal intelligence, auditory sequential memory, and language following right-sided tumors, and deficient nonverbal tasks including spatial and visual sequential memory and impaired prosody after left-sided tumors. Similar findings have been observed by others [184, 242, 243]. Behavioral changes can be marked and include disinhibition, impulsivity and irritability [244], dysphoria and inattention [237], anxiety, aggression [245], and stereotypes and aberrant interpersonal relations meeting criteria for the diagnosis of ASD [59].

Posterior fossa tumor resection in children can be complicated by the development of CB mutism [60, 246–253]. The consensus understanding of postoperative pediatric CB mutism syndrome [254] is that after a latent period of 1 to 2 days following CB or 4th ventricle tumor resection, children develop “mutism / reduced speech and emotional lability… Additional common features including hypotonia and oropharyngeal dysfunction / dysphagia. It may frequently be accompanied by the cerebellar motor syndrome, the cerebellar cognitive affective syndrome, and brainstem dysfunction including long tract signs and cranial neuropathies.” CCAS is thus the behavioral syndrome accompanying mutism, and it may persist after the transient period of mutism has resolved. Behavioral changes include regressive personality, apathy, and poverty of spontaneous movement. Emotional lability can be marked, with rapid cycling of emotional expression between irritability, inconsolable crying and agitation, to giggling and easy distractibility. CB mutism also occurs following stroke or hemorrhage in children [234, 255] and adults [256], and diminished verbal fluency approaching mutism was described in the original study following postinfectious cerebellitis [12].

### Developmental CCAS

Disruptions and gene disorders causing CB malformations result in a developmental form of CCAS. Children with CB hemorrhages in utero or early postnatal life have problems with expressive and receptive language and behavioral and social deficits that meet criteria for ASD in more than 40% [257, 258]. These observations of long-term sequela following CB hemorrhage in children have been confirmed by others [259]. Developmental CCAS was also observed in three brothers with hindbrain malformation in Joubert syndrome, a mutation in the TMEM67 gene [260]. The siblings were developmentally delayed, demonstrating disproportionate cognitive weaknesses in selected aspects of executive function, language processing, and the visuospatial domain, as well as a pronounced neuropsychiatric constellation. The long-term consequences of CB malformations on cognitive development may reflect a deficit in sustaining projections between CB and cerebral cortical and subcortical sites, functioning through trophic mechanisms required for the development and pruning of connections [2, 157, 258, 261, 262].

### Neuropsychiatry of the Cerebellum; the Affective Component of the CCAS

Emotional dysregulation can be prominent in CCAS [12, 60] and can be marked in children following tumor resection, as above. Patients with midline lesions demonstrate social–emotional aberrant behaviors [3, 12, 58, 60, 263], and social cognition is impaired in CB patients assessed using the “Reading the Mind in the Eyes” test [109]. CB lesions also affect encoding and processing of external negative stimuli [222] and conscious self-monitoring of negative emotion [264].

Opsoclonus myoclonus syndrome is a postinfectious or paraneoplastic immune-mediated phenomenon with a psychiatric constellation of mood changes, irritability, lability, aggression, and night terrors [265, 266]. Dysphoric mood, disinhibition and poor affect regulation, disruptive behaviors, and temper tantrums occur together with cognitive and language impairment [14, 266, 267].

Depression had a prevalence of 26% in the natural history study of 300 patients with SCAs 1, 2, 3, and 6, and suicidal ideation was present in 65% of the SCA3 patients [268]. CCAS is under active investigation in other psychiatric disorders including schizophrenia and ASD [3, 269–274]. CB lesions may dysregulate mood and personality and trigger psychotic thinking and behaviors that meet criteria for diagnoses of attention deficit hyperactivity disorder, obsessive–compulsive disorder, depression, bipolar disorder, ASD, atypical psychosis, anxiety, and panic disorder. These neuropsychiatric presentations have been conceptualized as emotional overshoot (hypermetria) or undershoot (hypometria) within five neuropsychiatric domains—attentional control, emotional control, social skill set, psychosis spectrum disorders, and ASD [14].

Single case studies provide the clinical relevance of these new approaches [14, 275]. This is exemplified by the recent report of a patient with rupture of a CB arteriovenous malformation who developed mania and personality and mood changes consistent with a borderline personality and bipolar I disorder [261]. The CB damage involved left lobules VI, VIIA-Crus I, and IX, and the posterior vermis. Altered functional connectivity was detected in prefrontal–striatal–thalamic circuits implicated in bipolar subjects during the manic state [276].

The CB role in ASD is an area of active investigation. Recent resting-state fMRI studies in ASD reveal altered functional connectivity between the dentate nucleus and the cerebral cortex [119] and decreased volume in right Crus II that correlates with the degree of autistic traits. Right Crus II is interconnected with contralateral frontal and temporal areas related to social cognition, and altered functional connectivity has been reported between the smaller Crus II and these cerebral areas [277]. In the Tsc1 (tuberous sclerosis) mouse model of ASD, neuromodulation of right Crus I (hemispheric extension of lobule VIIA) rescued social deficits, consistent with the suggestion that the dysfunction of cerebro-CB circuits underlies selected aspects of disrupted behavior in ASD [278]. It therefore appears likely that dysfunction reported within neural circuits engaged in social cognition in ASD is related, at least in part, to impaired interactions between focal CB regions and critical cerebral cortical nodes of the social brain.

### CCAS/Schmahmann Syndrome Scale

The diagnosis of the CCAS at the bedside or in the office has been a challenge, requiring comprehensive neuropsychological testing. This need was addressed by Hoche and colleagues [13], who studied 77 patients with CB disease, and 39 more in a validation cohort, to develop a brief battery of tests to identify the CCAS. This study reaffirmed the core executive, visual–spatial, linguistic, and affective features of the CCAS. It then used the data to derive the CCAS/Schmahmann Scale, a 10-min battery of cross-domain assessments to diagnose CCAS in patients with CB lesions with certainty. The new scale makes it possible to assess cognition and affect in CB patients, which is proving to be useful for both clinical and research purposes.

### Conclusion

Each aspect of the CCAS has been replicated in studies over the 20 years since its description. Ongoing studies of its executive, linguistic, visual–spatial, and affective components confirm each of these domains and provide new details about how they manifest in different disease states and at different ages. The introduction of the CCAS Scale and the adaptation of the tools of contemporary cognitive neuroscience to study the CB may add greater depth and complexity to the understanding of the CB role in cognition and emotion. They also hold promise for new approaches to the treatment of neurobehavioral/neuropsychiatric manifestations of CB disorders.

## Discussion: Consensus on CCAS and Future Directions (G.P.D. Argyropoulos, K. van Dun, and R.B. Ivry)

This paper gathered contributions from experts in the field of CB neurocognition, attempting to promote awareness of CCAS as a clinical entity and stimulate further research. The authors substantiated the concept of CCAS with recent evidence from different angles and examined current insight into rehabilitation. Several points of convergence were identified, with respect to both the interpretation of the findings in the literature and the outstanding questions for future research.

### Towards Voxel-Based Approaches

CCAS provides a clinical entity that lends support to a CB role in cognition and affect. Nevertheless, the majority of CB lesion-symptom mapping studies on nonmotor function have relied on single cases or patient cohorts divided according to crude grouping criteria. The introduction of VLSM in focal CB lesions, and VBM in degenerative CB disease, affords us the level of spatial precision (“Cerebellar Functional Topography in CCAS: Updates and Challenges,” “Cerebellar Neurocognition: Relevance to CCAS,” and “Replication of CCAS” sections) required to address the fact that cortico-CB functional networks do not abide by lobular boundaries. Such precision is also fundamental for symptom prediction and intervention planning (“Cerebellar Neurocognition: Relevance to CCAS” and “Introducing Cognition into Cerebellar Rehabilitation: Facts and Hopes**”** sections). Importantly, though, these methods require relatively large patient cohorts, and research would benefit from multi-center collaborations (“Cerebellar Functional Topography in CCAS: Updates and Challenges” section).

### Research on Social Cognition and Affect

The lack of sophisticated lesion-symptom mapping methods is particularly evident in the affective component of CCAS. This has been assessed in a less systematic fashion, with many studies employing clinical psychiatric assessment, with psychopathological diagnoses based on DSM. Moreover, there is no clear spatial segregation of neo-CB regions in terms of functional connectivity with limbic versus association cortices. Functional imaging of affective processing supports a CB role, but this is not confined to what is considered the limbic CB, i.e. the posterior vermis (“Cerebellar Functional Topography in CCAS: Updates and Challenges” section). Work on CB contributions to social cognition can inform further research on the CB and affect, although, similarly, imaging and stimulation studies on the mechanisms underlying impaired social action sequencing are currently lacking (“Cerebellar Neurocognition: Relevance to CCAS” and “Replication of CCAS” sections).

### Noninvasive Cerebellar Stimulation

Noninvasive stimulation, in particular (primarily tDCS and rTMS), is promising with respect to investigating cortico-CB connectivity and identifying causal relationships between CB function and behavior (“Cerebellar Neurocognition: Relevance to CCAS” section; but see [279] for concerns with replicability). Especially when combined with the newest imaging techniques, a better understanding can be obtained of the mechanisms underlying the symptoms, which can lead to a very specific goal-directed therapeutic approach (“Introducing Cognition into Cerebellar Rehabilitation: Facts and Hopes**”** section; see also [19]).

### Cerebellar Versus Cortical Cognitive Deficits

The identification of CCAS also highlights the importance of assessing patients with CB lesions in cognitive function, both at the acute stage and over the course of their rehabilitation (“Introducing Cognition into Cerebellar Rehabilitation: Facts and Hopes**”** and “Replication of CCAS” sections). Consistent with the modulatory CB role in nonmotor function, the cognitive deficits that may follow neo-CB lesions often reflect those observed after prefrontal damage, albeit in a milder fashion (“Cerebellar Functional Topography in CCAS: Updates and Challenges” and “Introducing Cognition into Cerebellar Rehabilitation: Facts and Hopes**”** sections). Further research is required to investigate whether CB-induced cognitive impairment should be treated in a manner distinct from deficits following cortical damage, and specific techniques may be required for rehabilitation (“Introducing Cognition into Cerebellar Rehabilitation: Facts and Hopes**”** section; see also [154]).

### From Functional Domains to Computations

The development of rehabilitation approaches would benefit substantially from identifying the particular CB computations across functional domains (“Introducing Cognition into Cerebellar Rehabilitation: Facts and Hopes**”** section). The question of relevance no longer pertains to the presence of a CB role in cognition and affect, but to the computation by which this is accomplished [2]. The term “dysmetria of thought” is, at present, descriptive, providing a broad characterization rather than specifying underlying mechanistic impairments. Various hypotheses have been put forth that might form the basis of the UCT, such as error-based learning, error monitoring, forward control, prediction, timing, or sequencing (“Cerebellar Neurocognition: Relevance to CCAS,” “Introducing Cognition into Cerebellar Rehabilitation: Facts and Hopes,**”** and “Replication of CCAS” sections). The variability in the cognitive deficits observed may be explained by the extent to which this CB computation is required in particular tasks [280]. Alternatively, the CB role in cognition and affect may be modulatory, helping coordinate processing within extra-CB structures. Identifying core computations will require a move beyond standard neuropsychological assessment, toward finer-grained behavioral paradigms.

### Beyond the “Motor Versus Cognitive” Dichotomy

Indeed, it may be argued that the distinctions between functional domains may obfuscate the computations performed. While distinct relationships have been identified between the domain of impairment and the localization of CB damage (“Cerebellar Neurocognition: Relevance to CCAS” and “Replication of CCAS” sections), we may still need to revise our methods for distinguishing between “motor” and “cognitive” function, open to the idea that CB function may require considering interfaces between higher-level function and motor-like operations (“Cerebellar Functional Topography in CCAS: Updates and Challenges” section).

### Broader Networks

Such network integration is of importance in considering symptom recovery and rehabilitation. Given the well-documented cerebro-CB structural and functional connectivity (“Cerebellar Functional Topography in CCAS: Updates and Challenges,” “Cerebellar Neurocognition: Relevance to CCAS,” and “Replication of CCAS” sections), the presence of diaschisis and the time course of its resolution need to be factored in for the prediction of symptom recovery and the development of rehabilitatory interventions (“Introducing Cognition into Cerebellar Rehabilitation: Facts and Hopes**”** section). Similarly, understanding the time course of different symptom clusters could offer clues concerning their direct (CB) or indirect origins (off-target effects). Indeed, very little is known on the way in which the behavioral consequences of CB damage are modulated by extra-CB pathology. This network-based approach should be extended beyond cortico-CB circuits, to incorporate findings on the structural and functional connectivity of the CB with the BG and the medial temporal lobe (“Cerebellar Functional Topography in CCAS: Updates and Challenges” section). Longitudinal follow-up studies of CB patients, investigating behavioral improvement linked to the results obtained with structural and functional imaging techniques, might provide further insight into the CB role and the compensatory mechanisms after deprivation of CB–cerebral communication.

### Conclusion

CCAS reflects a constellation of CB-induced sequelae in executive function, visuospatial cognition, language, and emotion/affect. As a clinical entity, it supports a CB role in cognition and affect and provides ideal grounds for the investigation of CB contributions in nonmotor functions. Enhanced symptom prediction, intervention planning, and rehabilitation will benefit from (i) patient studies employing finer-grained behavioral paradigms that could identify the CB processes involved in cognition and affect, (ii) sophisticated lesion-symptom mapping methods to highlight intra- and cross-lobular regions in relation to particular symptoms, and (iii) research on the indirect effects of focal CB lesions on behavior by off-target effects and broader network dysfunction.

## Footnotes

1 The CB cortex comprises ten lobules; the anterior lobe is formed of lobules I–V; the posterior lobe comprises lobules VI–IX; lobule X is identified as the flocculonodular lobe. We follow the nomenclature in the “MRI Atlas of the Human Cerebellum” [29]: CB lobules are labeled “I–X” from the anterior–superior border, through posterior–superior, posterior–inferior, to the anterior–inferior border. The nomenclature is based on that of Larsell and Jansen [30]—hemispheres are distinguished from the vermis with the “H” prefix. The atlas of Schmahmann and colleagues [29] uses this prefix to refer to the hemispheres, and the adjective “vermal” to refer to vermal compartments of a lobule. No prefix is used when referring to the entire lobule.

2 The term “lesion-symptom mapping” is used to denote a broader range of methods used to investigate the relationship between symptoms and lesions, not necessarily in a voxel-based fashion, whereas the term “voxel-based lesion-symptom mapping” (“VLSM”) is reserved for the subset of these methods employing a voxel-based approach.
